# A Clinical Assessment of a Magnetic Resonance Computer-Aided Diagnosis System in the Detection of Pathological Complete Response After Neoadjuvant Chemotherapy in Breast Cancer

**DOI:** 10.3389/fonc.2022.784839

**Published:** 2022-03-03

**Authors:** Haiyong Peng, Shaolei Yan, Xiaodan Chen, Jiahang Hu, Kaige Chen, Ping Wang, Hongxia Zhang, Xiushi Zhang, Wei Meng

**Affiliations:** ^1^ Radiology Department, Harbin Medical University, Harbin Medical University Cancer Hospital, Harbin, China; ^2^ Department of Computer Technology, Harbin Institute of Technology University, Harbin, China; ^3^ Department of Radiology, Hongqi Hospital Affiliated to Mudanjiang Medical University, Mudanjiang, China

**Keywords:** breast cancer, MRI, computer-aided diagnosis, pathological complete response, neoadjuvant chemotherapy (NAC)

## Abstract

**Purpose:**

This study aimed to assess the diagnostic performance and the added value to radiologists of different levels of a computer-aided diagnosis (CAD) system for the detection of pathological complete response (pCR) after neoadjuvant chemotherapy (NAC) in patients with breast cancer. Besides, to investigate whether tumor molecular typing is associated with the efficiency of diagnosis of the CAD systems.

**Methods:**

470 patients were identified with breast cancers who underwent NAC and post MR imaging between January 2016 and March 2019. The diagnostic performance of radiologists of different levels and the CAD system were compared. The added value of the CAD system was assessed and subgroup analyses were performed according to the tumor molecular typing.

**Results:**

Among 470 patients, 123 (26%) underwent pCR. The CAD system showed a comparable specificity as the senior radiologist (83.29% vs. 84.15%, p=0.488) and comparable area under the curve (AUC) (0.839 vs. 0.835, p =0.452). The performance of all radiologists significantly improved when aided by the CAD system (P<0.05), And there were no statistical differences in terms of sensitivity, specificity and accuracy between the two groups with CAD assistance(p>0.05).The AUC values for identifying pCR in TN patients were significant (0.883, 95%CI: 0.801-0.964, p < 0.001).

**Conclusion:**

The CAD system assessed in this study improves the performance of all radiologists, regardless of experience. The molecular typing of breast cancer is potential influencer of CAD diagnostic performance.

## Introduction

With the wide application of neoadjuvant chemotherapy in the treatment of breast cancer patients, it has become an essential part of the treatment of breast cancer, especially stage II and III breast cancer (1, 2). Its curative effect directly affects the follow-up treatment and prognosis of patients. Effective NAC can reduce tumor stage, make breast conserving surgery possible, and even achieve preoperative pathological complete remission (pCR) in up to 30% of patients (3, 4). The efficacy of chemotherapy varies and depends on the subtypes of breast cancers (5). HER2- positive and triple-negative patients are more likely to achieve pCR, and surgery is expected to be avoided (6). As a consequence, accurate recognition of treatment response is crucial to optimize patient management and treatment adjustment.

Conventional imaging modalities, such as mammography and ultrasound, show limited accuracy in predicting treatment response after NAC (7, 8), Magnetic Resonance Imaging (MRI) is currently used in clinical practice to assess the response at the end of NAC. Several studies have investigated the value of breast MRI for assessing or predicting treatment response to NAC (9–11). However, MRI has limitations when used clinically because image interpretation is based on the radiologist’s visual assessment.

Computer-aided diagnosis (CAD) has attracted significant attention from researchers as a newly developed technique that can enhance radiologists’ interpretation and overcome subjective limitations (12–15). The CAD detection and diagnosis methods are based on machine learning approaches that extract features based on shape, texture, and statistical values, assessing or predicting treatment response to NAC. Several studies have shown that the CAD system has superior capability and performance (16, 17). However, few studies have evaluated the changes in diagnosis performance when the CAD system combined with radiologists with various levels of experience in assessing response to chemotherapy after treatment.

Therefore, this retrospective study aimed to validate the clinical role of the CAD systems in the assessment of pCR and to evaluate its value in improving doctors’ diagnosis performance. Besides, the association between the efficiency of diagnosis of the CAD systems and tumor subtypes was discussed.

## Materials And Methods

**Figure 1:**
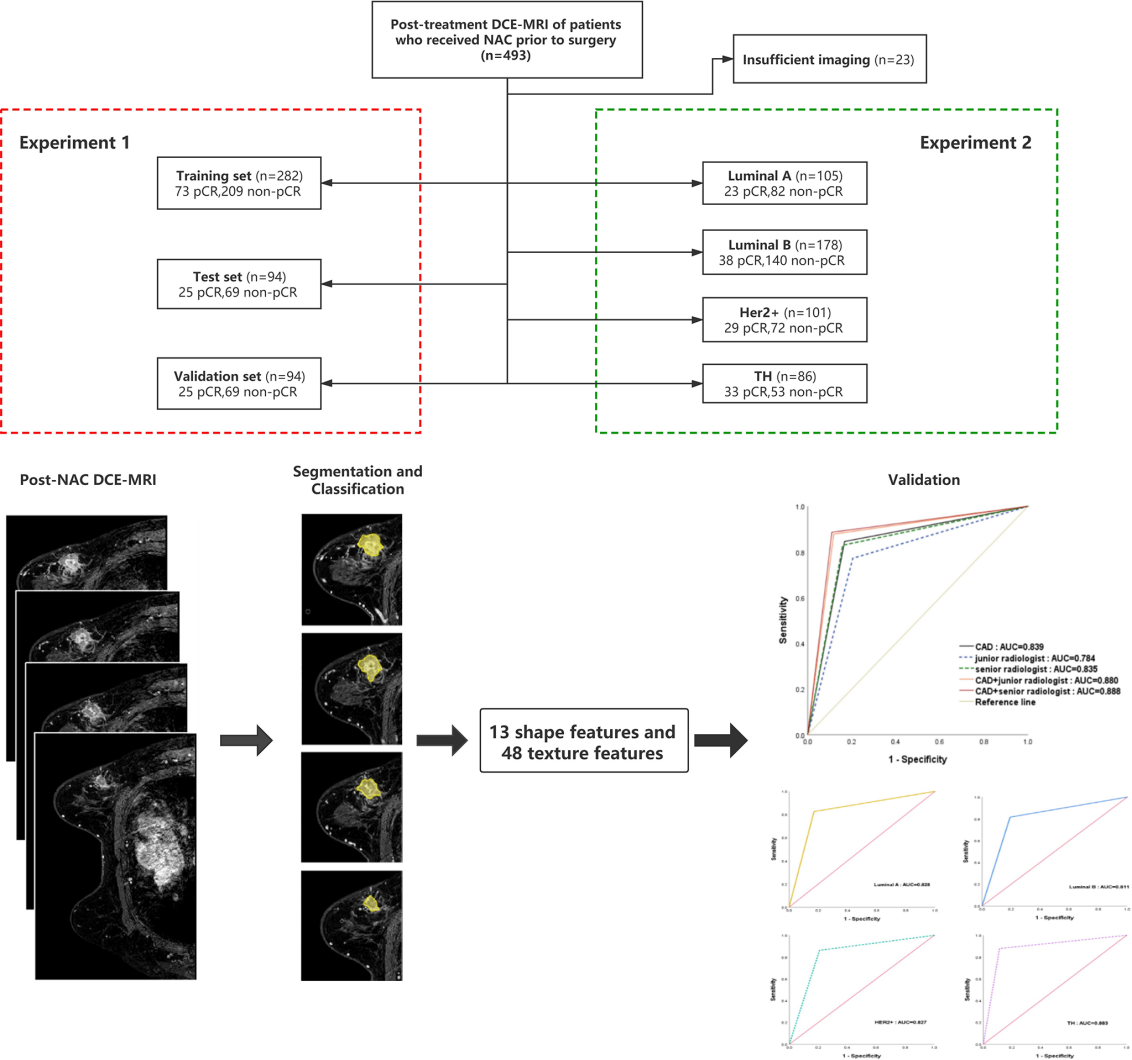
Patients selection flowchart and the composition of the training, test, and verification sets. pCR, pathologic complete response; NAC, neoadjuvant chemotherapy.

The institutional review board approved this retrospective study. Informed consent was obtained from all the patients. All patients in whom invasive breast cancer were diagnosed between January 2016 and March 2019, treated with neoadjuvant chemotherapy, and who underwent breast MR imaging before neoadjuvant chemotherapy were eligible. The chemotherapy regimens were drawn up according to the neoadjuvant therapy regimens of NCCN guidelines breast cancer version 1.2016 including (regimen I): AC-T(doxorubicin 60mg/m2 plus cyclophosphamide 600mg/m2 IV day 1 every 21 days for 4 cycles followed by docetaxel 100mg/m2 IV day 1 every 21 days for 4 cycles); (regimen II):TAC(docetaxel 75mg/m2 plus doxorubicin 50mg/m2 plus cyclophosphamide 500mg/m2 every 21 days for 6 cycles). Trastuzumab or Partuzumab would employ in HER2/neu positive patients (Trastuzumab:the dose was 4mg/kg for the first use; the followed dose was 2mg/kg, i.e., every 21 days for 1 year; Partuzumab: the dose was 840mg/kg for the frst use; the followed dose was 420mg/kg, i.e., every 21 days for 1 year). A total of 493 patients (mean age: 49.6 ± 10.09 years; range: 24-70 years) and 470 masses (mean size before chemotherapy: 19.03 ± 7.1mm; range: 6-55mm) underwent core needle biopsy or surgery. Twenty-three patients were excluded from the study group, because the patient had unilateral multifocal cancers and the correlation between the tumor in MRI and postoperative pathological examination was uncertain. A flowchart of the study population is presented in **Figure 1**.

MR images were obtained using a 3.0T MR scanner (Philips Achieva 3.0T). The patients adopted a prone position and put their breasts into the dedicated phased-array breast coil. Imaging parameters for DCE-MRI were are as follows:

Axial T1-weighted imaging (repetition time (TR) = 495 ms; echo time (TE) = 10 ms; slice thickness/gap = 3 mm/0 mm; matrix = 512; number of signal averaged (NSA) = 1; field of view (FOV) = 340 mm × 340 mm); axial T2-weighted imaging (TR = 4213 ms, TE = 120 ms, slice thickness/gap = 3 mm/0 mm, matrix = 512, NSA = 1, FOV = 340 mm × 340 mm); T2-weighted fat-saturated imaging using a spectral selection attenuated inversion recovery (SPAIR) (TR = 4216 ms, TE = 60 ms, inversion delay (IR) = 120 ms, slice thickness/gap = 3 mm/0 mm, matrix = 352, NSA = 1, FOV = 340 mm × 340 mm); and T1-weighted high-resolution isotropic volume examination (THRIVE) (TR = 4.4 ms, TE = 2.2 ms, flip angle = 12°; matrix = 352; FOV = 340 mm × 340 mm; number of sections = 110; acquisition time: 256 seconds). MR imaging data sets were acquired once before gadolinium (Gd)- diethylenetriamine penta-acetic acid (DTPA) (Bayer scheming pharma AG, Berlin, Germany) injection and at 90-second intervals upon injection of 0.1 mmol/kg Gd-DTPA(followed by an intravenous saline flush of 20 ml), for a total imaging duration of 5–8 minutes.

### Segmentation and Classification

**Figure 2:**
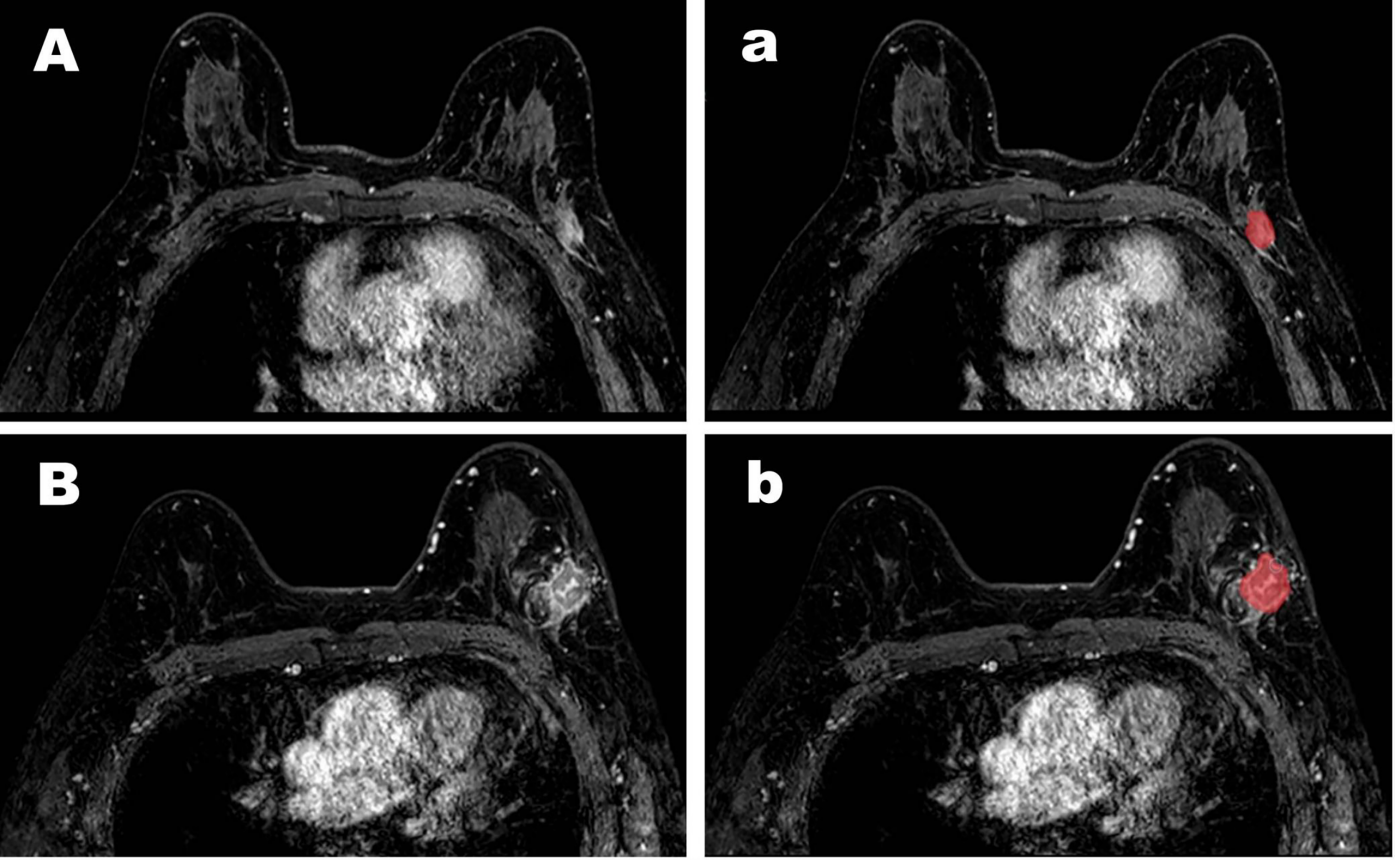
Representative cases of pCR **(A)** and non-pCR **(B)**. For the case **(A)**, both the CAD system and the senior radiologists diagnosed it as a pCR but the junior radiologists diagnosed it as a non-pCR. For the case **(B)**, both the CAD system and the senior and the junior radiologists diagnosed it as a non-pCR. The images (a, b) for the segmentation results were obtained by computer-aided diagnosis system.

We first used an encoder-decoder network called Unet to segment the tumor region in the MRI, shown in **Figure 2**. The encoder network in Unet extracts the deep semantic features in MRI, and the decoder network upsamples the features to the size of the original image. The backbone of the encoder is resnet18, and the strategy of the decoder is upsampling step by step. The learning rate of training is 1e-5, and epochs are 500. The weight decay is 5e-4 and the training optimizer is Adam. The loss function is Cross Entropy. Thus, the Unet model segment the tumor region from the background. And then we extracted shape features and texture features of tumor. The 13 shape features describe the appearance of tumor, which include roundness, aspect ratio, average normalized radial length, the normalized standard deviation of radial length, average normalized entropy radial length, area ratio, aspect ratio, number of leaflets, needle shape, boundary roughness, direction angle, normalized ellipse circumference and normalized ellipse contour. The 48 texture features show the details inside tumors obtained using gray level co-occurrence matrix (GLCM). Moreover, we extracted energy, correlation, contrast and entropy under three steps with four directions. The 13 shape features and 48 texture features were input into the support vector machine to execute pCR or non-pCR classification. The goal of Support Vector Machine (SVM) is to find a hyperplane to separate the two classes of data and maximize the margin in the meantime. The data which is closest to the margin is called a support vector and the distance between the hyperplane and any support vector is 1.

### Observer Study

The MR images were assessed by a senior radiologist of more than ten years’ experience and then assessed by a junior radiologist of three years’ experience. The two groups of radiologists analyzed the integrated computer workstation images without access to the final histological results. The diagnosis of the pCR was based on whether the tumor volume disappeared or marked and constantly homogeneous enhancement fibrous tissue on DCE-MRI. Only the largest mass was used for evaluation if a patient had multiple residual masses after NAC. If there was disagreement between the two radiologists, they reviewed the images together, obtaining a consensus.

### Pathological Diagnoses

All breast lesions were pathologically confirmed by surgery or biopsy. Pathological complete remission (pCR) was defined as no residual invasive tumor cells in primary breast lesions after therapy, but ductal carcinoma in situ(DCIS) can exist. Lesions were divided into pCR and non-pCR groups, and all the lesions were divided into molecular subtypes. All the assessments were performed by a pathologist who had more than ten years’ experience. Tumor characteristics are presented in **Table 1**.

**Table 1:** Breakdown of dataset by pathological complete response status.

	pCR	Non-pCR	All patients
**Number of patients**	123 (32–66)	347 (24–70)	470 (24–70)
Mean Age (y)*	54	48	50
Tumor diameter (mm)*			
Mean	22.1	32.2	29.0
SD	12.5	13.9	14.3
**Receptor status**			
Luminal A	23	82	105
Luminal B	38	140	178
HER-2+	29	72	101
TN	33	53	86
**Surgery type**			
Breast conservation	114	218	332
Mastectomy	9	129	138

*Data are means, with ranges in parentheses.

### Statistical Analysis

The diagnostic performance of the radiologist assisted by the CAD system was defined as positive when the criteria met one of the two categories: the radiologist and the CAD system. The SPSS software (version 20.0, IBM Corp, Armonk, NY, United States) and MedCalc software (version 15.2, Mariakerke, Belgium) were used to analyze the data. Taking molecular subtypes as the standard, the separate diagnostic ROC curves of luminal a, luminal B, HER2 +, TN were constructed; The ROC curves for the separate diagnosis of junior radiologist, senior radiologist and CAD and the joint diagnosis of junior radiologist and CAD, senior radiologist and CAD were constructed by comparing the pathological results. and the area under the curve (AUC) and sensitivity, specificity and accuracy were calculated. Chi square test was used to compare the sensitivity, specificity and accuracy of different diagnostic methods. Inspection level α=0.5.

## Results

There were pCR and non-pCR in the 493 patients (mean age: 49.6 ± 10.09 years; range: 24-70 years). The experimental data were 470 MRI masses (average size before NAC: 19.03 ± 7.1 mm, range:6-55mm), of which 347(74%) were non-pCR, and 123(26%)were pCR. The non-pCR images and pCR images were divided into 5 parts respectively. Each time, 3 parts were taken as the training set, 1 part as the verification set and 1 part as the test set.

The diagnostic performances of the CAD system, radiologists in the different groups, and CAD-assisted radiologists for detecting pCR were summarized in **Table 2**.

**Table 2:** Diagnostic performance of CAD system, radiologists and CAD-assisted radiologists.

Method	AUC	95%CI	Sensitivity	Specificity	Accuracy
Junior radiologist	0.784	0.734-0.833	77.24	79.54	78.94
Senior radiologist	0.835	0.791-0.880	82.93	84.15	83.83
CAD	0.839	0.796-0.883	84.55	83.29	83.61
Junior radiologist+CAD	0.880	0.841-0.919	87.80	88.18	88.09
Senior radiologist+CAD	0.888	0.851-0.926	88.62	89.04	88.94
P^a1^	0.049		<0.001	0.007	<0.001
P^a2^	0.452		0.005	0.488	0.037
P^b1^	0.001		<0.001	<0.001	<0.001
P^b2^	0.037		<0.001	0.001	<0.001
P^*^	0.380		0.525	0.713	0.525

P^a1^ is CAD vs. Junior radiologist; P^a2^ is CAD vs. Senior radiologist; P^b1^ is Junior radiologist vs. Junior radiologist+CAD.

P^b2^ is Senior radiologist vs. Senior radiologist+CAD; P^*^ is Junior radiologist+CAD vs. Senior radiologist+CAD.

**Figure 3:**
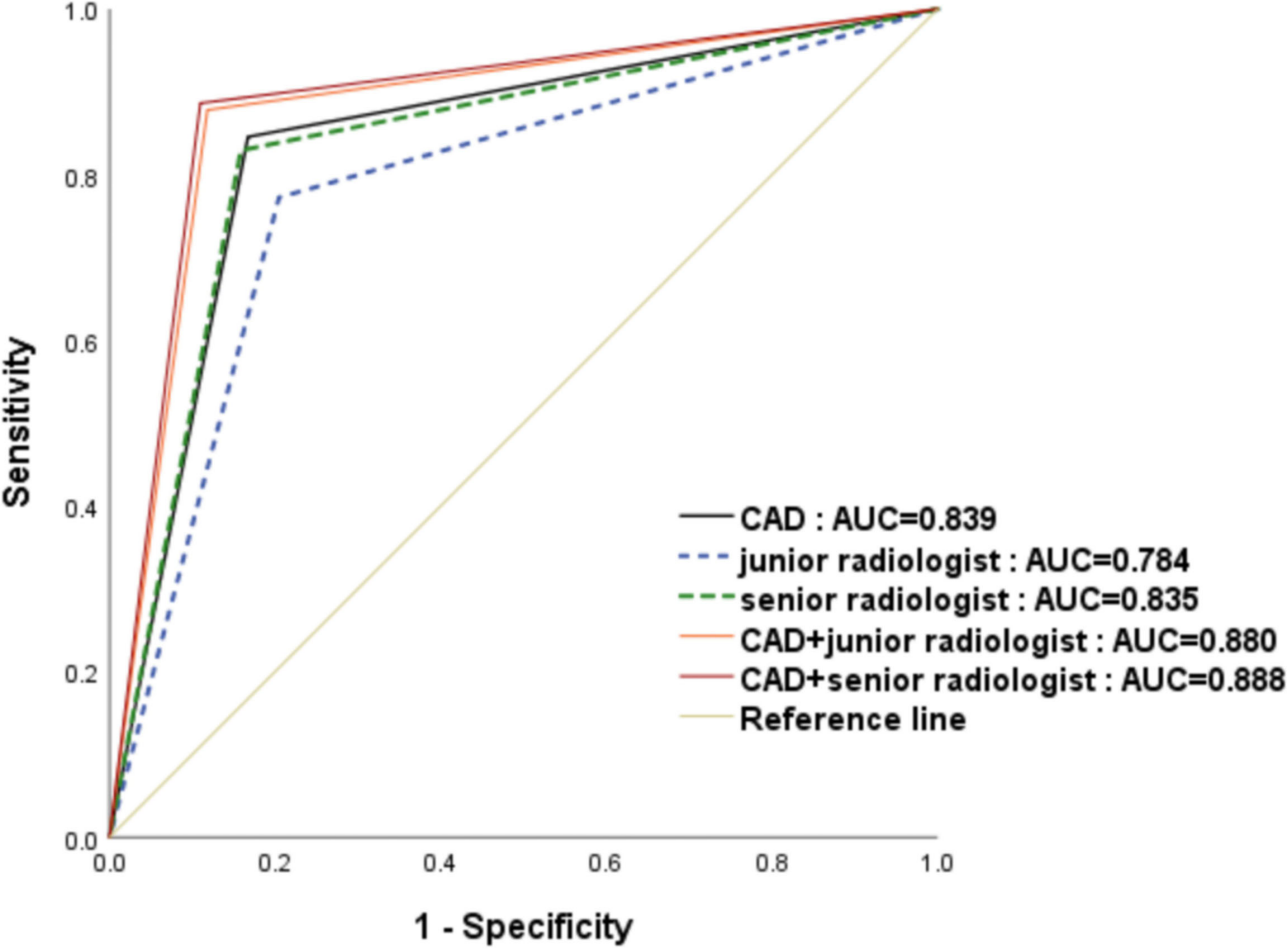
The receiver operating characteristic (ROC) curves for the performance of the computer-aided diagnosis (CAD) system, the senior radiologist, the junior radiologist, and CAD-assisted radiologists. The area under the ROC curve for the combination of senior radiologists and CAD was significantly highest.

The CAD system exhibited no statistically significant difference in terms of specificity compared with the senior radiologist(83.29% versus 84.15%,p=0.488),and CAD has higher sensitivity while the accuracy were lower in the CAD system than those in the senior radiologist(84.55% vs. 82.93%,p=0.005;83.61% vs. 83.83%,p=0.037,respectively). When compared with the junior radiologist, the CAD system resulted in markedly increased sensitivity and accuracy and higher specificity in the classification of pCR (84.55% vs.77.24%, p <0.001; 83.83% vs.78.94%, p<0.001; 83.29% vs.79.54%, p = 0.007, respectively). When the CAD system was used to assist the senior and junior radiologists, the sensitivity, specificity and accuracy of diagnosis were significantly improved, no matter junior radiologist or senior radiologist(p ≤ 0.001).And there was no statistical difference terms of sensitivity, specificity and accuracy between the two groups with CAD assistance(87.80% vs.88.62%, p =0.525; 88.18% vs.89.04%,p=0.713; 88.94% vs.88.09%, p = 0.525, respectively). ROC analysis comparing the diagnostic performance of CAD systems, radiologists, and CAD-assisted radiologists is shown in **Table 2** and **Figure 3**. The AUCs were 0.784 for the junior radiologist,0.835 for the senior radiologist,0.839 for the CAD system, 0.880 for the CAD-assisted junior radiologist,0.888 for the CAD-assisted senior radiologist.

**Figure 4:**
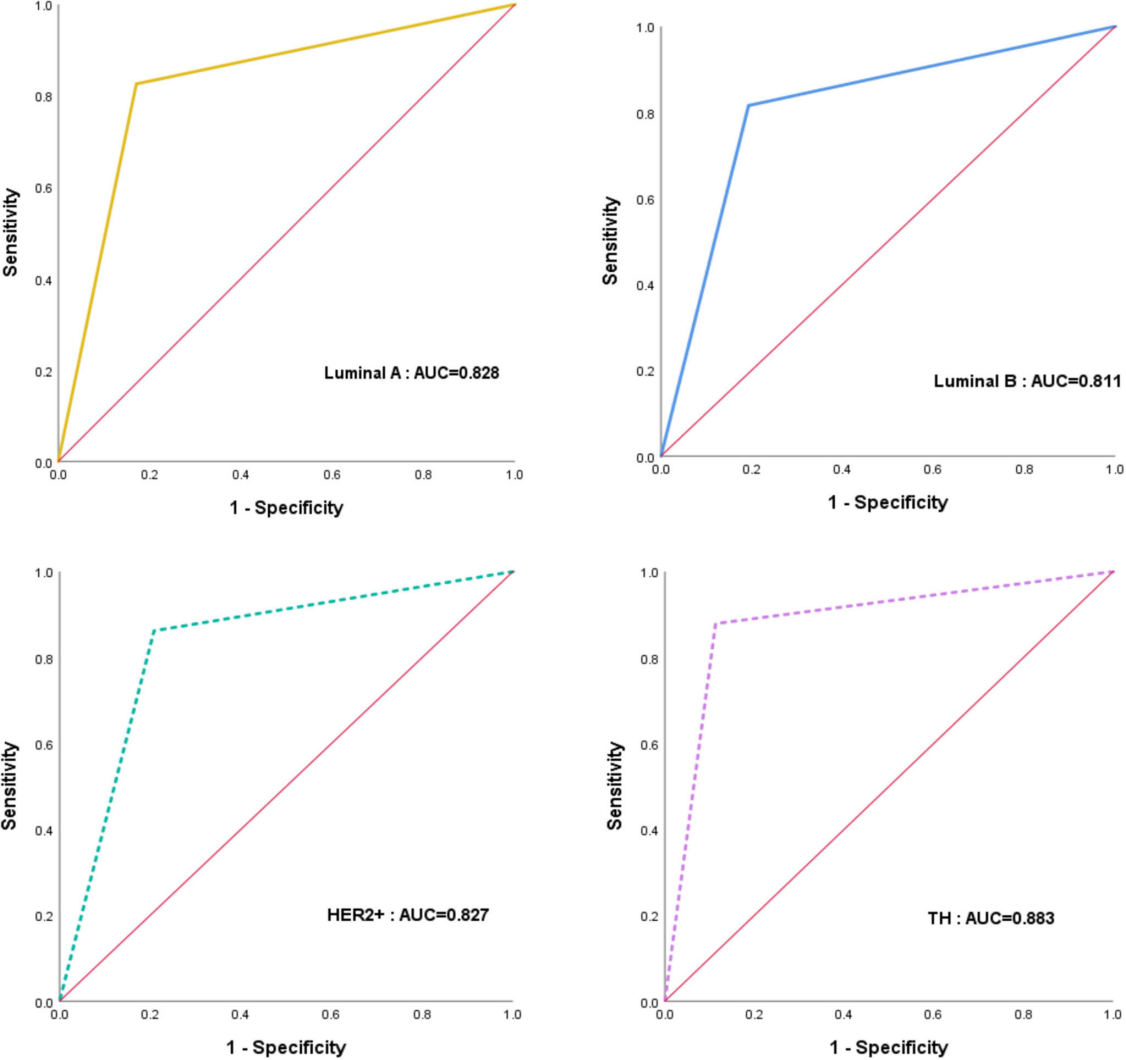
The receiver operating characteristic (ROC) curves for the performance of CAD in different molecular subtypes.

Results of performance of CAD in different molecular subtypes are listed in **Table 3** and **Figure 4**. Out of the 123 patients who achieved pCR, twenty-three breast cancers were luminal A, thirty-eight were luminal B, twenty-nine were HER2-enriched, and thirty-three were triple-negative. The AUC values for identifying pCR in TN patients were significant (0.883, 95%CI: 0.801-0.964, p < 0.001), and the specificity, sensitivity and accuracy achieved 88.68%, 87.88% and 88.37%, respectively.

**Table 3:** Diagnostic efficacy of the diagnosis of CAD among subtype.

All patients	AUC	95%CI	P	Sensitivity	Specificity	Accuracy
**Luminal A**	0.828	0.726-0.929	<0.001	82.61	82.93	82.86
**Luminal B**	0.811	0.731-0.892	<0.001	81.58	80.71	80.90
**HER2+**	0.827	0.736-0.918	<0.001	86.20	84.72	85.15
**TN**	0.883	0.801-0.964	<0.001	87.88	88.68	88.37

## Discussion

MR plays a crucial role in the assessment of response to chemotherapy during treatment. However, the usefulness of MR may be limited for the diagnostic performance of it varies from person to person, which depends on the experience of a radiologist to a large extent. The CAD system based on artificial intelligence has been developed to assist radiologists in analyzing images, shortening the time cost of the diagnostic process, and reducing interobserver variability.

In this study, a clinical assessment was performed to evaluate the value of the CAD system in the MRI diagnosis of pCR. This retrospective study showed that the CAD system generally performed comparably to qualitative assessments by the senior radiologist in terms of specificity but had a higher sensitivity and lower accuracy. In addition, the specificity, sensitivity and accuracy of the CAD system were remarkably higher than that of the junior radiologist.

The added value of the CAD system was also evaluated in this study. Our study showed that CAD assistance significantly improves all radiologists’ performance, which was consistent with some studies (12, 18, 19). With the assistance of the CAD system, the junior radiologist showed a significant increase in AUC from 0.784 to 0.880(P<0.001). The diagnostic performance of senior radiologists was also improved and statistically significant(P<0.05). The improved AUC indicated that the CAD system might function as a supplementary opinion to avoid missed diagnoses, especially for less-experienced radiologists. As shown in the study, the CAD system improved radiologist specificity, which implied that the CAD system could play a constructive role in reducing unnecessary biopsies or follow-up imaging studies to assess response to chemotherapy.

The study contributes to several clinical implications. First, the CAD system in this study can automatically recognize and analyze MR images. Therefore, it is also possible to overcome the disadvantages caused by the visual assessment of radiologists, which demonstrates an opportunity for the combination between radiologists and machines in future clinical practice. Second, the CAD system exhibited no statistically significant difference in specificity compared with the senior radiologist. In addition, the sensitivity and accuracy were higher. This finding implied that the CAD system could reduce unnecessary biopsies and also help to lighten the load of radiologists. Besides, all individual radiologists significantly improved with CAD assistance, which could serve as a supplementary diagnosis for radiologists to minimize missed diagnoses, Especially for inexperienced radiologists. Lastly, the CAD system’s diagnostic efficiency for assessing response to chemotherapy during treatment was evaluated, which further reflected the clinical value of the CAD system.

We further analyzed whether the efficiency of diagnosis of the CAD systems was affected by molecular typing. In previous studies, Cain developed a multivariate machine learning model using 288 pre-NAC MRIs. They found that this model was significantly associated with pCR in TN/HER2 + patients, reaching an AUC of 0.707 (20). Braman also identified that the TN/HER2 + combined tumor subtype could predict pCR more accurately than the HR and HER2 + tumor subtypes (AUC = 0. 93) by extracting intratumoral and peritumoral features (21). However, they grouped TN and HER2+ patients into a combined TN/HER2+ cohort because of insufficient sample sizes. Moreover, they used the pre-NAC MRI images, which is different from our study. One of our methodologies vital advantages was that our experiments utilize computers to process segmentation, classification, and subtyping of tumors simultaneously. Moreover, we extracted 13 shape features and 48 texture features of tumor to improve the classification. In summary, TN cancers seemed to carry distinct radiomic signatures that enable CAD to separate from breast cancers with other features. One possible explanation for the findings may be that the TN subtypes demonstrated more necrosis so the texture may be more features in the images.

This study also has some limitations. First of all, the sample capacity was relatively small, and selection bias was inevitable due to the retrospective study nature. Therefore, additional studies with a more significant number of NAC cases are required to establish the clinical value of CAD in predicting the pCR after NAC. Second, the MRI scans we used were only two-dimensional rather than three-dimensional. So, it may not have represented the entire tumor exactly. Finally, no formal training for the processed images was used in our study. Although the processed images’ features were familiar to the radiologists, a training set to allow radiologists to become familiar with the CAD method might enhance their confidence to use it.

In conclusion, the CAD system assessed in this study improves the performance of all radiologists, regardless of experience, in classifying pCR on MRI. The molecular typing of breast cancer is a potential influencer of CAD diagnostic performance. Future work will address using a larger independent dataset for testing to improve its diagnostic performance and evaluate the clinical role of CAD diagnosis. CAD systems may improve the specificity of MRI and yield high clinical impact, especially among radiologists with limited experience in MRI.

## Data Availability Statement

The raw data supporting the conclusions of this article will be made available by the authors, without undue reservation.

## Ethics Statement

Written informed consent was obtained from the individual(s) for the publication of any potentially identifiable images or data included in this article.

## Author Contributions

HP and SY created the datasets, interpreted the data and defined the clinical labels. XC, XZ, HZ, PW, KC, and JH developed the network architecture and training and testing setup. HP and SY created the figures and performed statistical analysis. HP wrote the manuscript. WM provided the clinical expertise and guidance on the study design. WM supervised the project. All authors contributed to the article and approved the submitted version.

## Funding

The work was supported by the National Natural Science Foundation of China (81901701), the Postdoctoral Science Foundation of the Ministry of Heilongjiang Province(LBH-Q20123)and Harbin Medical University Cancer Hospital Haiyan fund (JJMS2021-31, JJZD2021-15).

## Conflict of Interest

The authors declare that the research was conducted in the absence of any commercial or financial relationships that could be construed as a potential conflict of interest.

## Publisher’s Note

All claims expressed in this article are solely those of the authors and do not necessarily represent those of their affiliated organizations, or those of the publisher, the editors and the reviewers. Any product that may be evaluated in this article, or claim that may be made by its manufacturer, is not guaranteed or endorsed by the publisher.
